# Anorectal and urogenital functional outcome after robotic and transanal total mesorectal excision for rectal cancer: a propensity score-matched analysis

**DOI:** 10.1007/s10151-025-03172-w

**Published:** 2025-07-14

**Authors:** Pak Chiu Wong, Felix Che Lok Chow, Wai Lun Law, Chi Chung Foo

**Affiliations:** 1 Division of Colorectal Surgery, Department of Surgery, Queen Mary Hospital, 102 Pokfulam Road, Hong Kong, China; 2 Division of Colorectal Surgery, Department of Surgery, School of Clinical Medicine, The University of Hong Kong, Hong Kong, China

**Keywords:** Rectal cancer, Robotic total mesorectal excision, TaTME, Low anterior resection syndrome, Fecal incontinence, Urogenital function

## Abstract

**Background:**

Robotic-assisted total mesorectal excision (RaTME) and transanal TME (TaTME) are well-established approaches for rectal cancer with promising oncological outcomes. Concerns about postoperative defecatory, urinary, and sexual dysfunction have been raised and the impact on patients’ quality of life remained uncertain. This study compared anorectal and urogenital functional outcomes after RaTME and TaTME.

**Methods:**

Patients with mid to low rectal cancer who underwent sphincter-saving surgery between January 2016 and December 2021 were reviewed. Questionnaires regarding low anterior resection syndrome (LARS), Wexner incontinence score, International Prostate Symptom Score (IPSS), and International Index of Erectile Function (IIEF-5) were administered at 1, 3, 6, and 12 months after index operation without stoma or after stoma closure.

**Results:**

Two hundred patients were included with 108 and 92 patients in the RaTME and TaTME group, respectively. After matching, 74 patients were analyzed in each group. LARS scores were significantly lower in the RaTME group than the TaTME group at 6 months (27 [interquartile range (IQR) 13–36] vs 30 [IQR 24–39], *p* = 0.038) but similar at 12 months (27 [IQR 13–33] vs 29 [IQR 13–36], *p* = 0.369) after stoma closure. Urinary function deteriorated after both operations but recovered at 6 months after RaTME and 12 months after TaTME. For sexual function, IIEF scores remained similar in the two groups.

**Conclusion:**

RaTME provided better anorectal function with lower LARS score at initial postoperative 6 months but similar after 1 year. Urinary function recovered earlier at 6 months after RaTME while sexual function was comparable between two groups.

## Introduction

Total mesorectal excision (TME) has become the gold standard surgical treatment for rectal cancer since its proposal in 1982 [1]. It can be either performed by a top-down approach or by a bottom-up approach with transanal platform [2]. Over the last decade, the top-down or transabdominal approach has evolved from conventional open and laparoscopic TME to robotic assisted TME (RaTME) owing to the advantage of improved instrument dexterity and three-dimensional magnified view [3]. On the other hand, transanal TME (TaTME) has become the alternative and widely adopted approach with its unique superiority to obtain precise dissection plane from distal rectum and perform ultralow anastomosis [4, 5].

With a standardized technique to preserve the mesorectal fascia during TME, oncological outcomes of both approaches were proven to be promising in reducing local recurrence [6, 7]. However, radical dissection along the mesorectal fascial plane may lead to inadvertent injury to hypogastric nerve plexus and neurovascular bundle in the pelvis [8]. The resultant significant impact on defecatory, urinary, and sexual function may compromise patient’s quality of life [9, 10].

In recent years, functional outcome of rectal cancer surgery has become an important aspect to be studied. However, the few non-randomized studies demonstrated conflicting results between robotic and transanal TME [11, 12]. The objective of this study is to compare anorectal and urogenital functional outcome in patients with rectal cancer treated by these two approaches.

## Methods

### Patient selection

All consecutive adult patients with mid to low rectal cancer who received elective sphincter-saving operation in our center between January 2016 and December 2021 were reviewed. TME performed with curative intent via robotic assisted laparoscopic approach (RaTME) or transanal approach (TaTME) were included. Exclusion criteria were patients with concomitant colectomy, open surgery, partial mesorectal excision, history of urological operation and pelvic irradiation for other malignancy.

### Surgery and oncological treatment

The standardized protocol for preoperative assessment, neoadjuvant treatment, and surgical techniques were described in previous publications [13, 14]. In short, all patients with newly diagnosed rectal cancer were discussed in a multidisciplinary meeting with oncologists and radiologists. Upon review of magnetic resonance imaging findings, patients with stage T3c or above, node-positive, threatened, or involved mesorectal fascia, and positive extramural venous invasion were referred to an oncologist for neoadjuvant long-course chemoirradiation. Surgical approach for TME was decided on the basis of tumor height measured as the distance away from the anal verge, pelvic anatomy, and surgeon’s preference. In RaTME, the robotic system was docked and dissection was started with high ligation of inferior mesenteric vessels followed by mobilization of sigmoid and rectum down to pelvic floor. In TaTME, a two-team approach was adopted in which the abdominal part was performed in laparoscopic manner and the transanal dissection was performed using a transanal access platform—GelPOINT Path (Applied Medical, Rancho Santa Margarita, CA). Mechanical bowel preparation with polyethylene glycol was prescribed to patients without clinical sign of tumor obstruction. Diversion stoma was constructed for anastomotic height within 6 cm from the anal verge.

Postoperative care was standardized according to the enhanced recovery after surgery pathway in our unit. Stoma reversal was arranged as soon as the patient recovered from the index operation or after completion of adjuvant chemotherapy when deemed necessary. Bowel training and education was delivered by a colorectal nurse specialist. Anal sphincter strengthening exercises and lifestyle modification were advised.

### Functional assessment

The primary outcome was the anorectal and urogenital functional outcome assessed by four validated questionnaires regarding LARS [15], Wexner incontinence score [16], IPSS [17], and IIEF-5 [18]. Scores were collected prospectively during preoperative assessment and regular postoperative follow-up visits. For anorectal function, assessment was performed at 1, 3, 6, and 12 months after index operation if no stoma was constructed or after stoma closure whichever was later. Urinary and sexual functions were assessed in male patients at the aforementioned time intervals after index operation.

### Statistical analysis

Patient demographics and clinical and pathological data were retrieved from the hospital computed management system and retrospectively reviewed. Propensity score matching using nearest-neighbor method with 1:1 caliper and matching tolerance of 0.1 was performed with tumor height and use of neoadjuvant irradiation being adjusted. Mann–Whitney *U* test was performed for analysis of functional scores between two groups. Paired-sample *t* test was used for comparison of functional scores across the follow-up period within each group. A *p* value of < 0.05 was considered statistically significant. SPSS version 25 was used for statistical analysis. Continuous variables were expressed as median with interquartile range (IQR).

## Results

Two hundred patients with rectal cancer were eligible for analysis during the study period including 108 and 92 patients in the RaTME and TaTME groups, respectively. Median age was 66 (IQR 59–72) and 78% of patients were male. The median follow-up time was 48 months (IQR 30–69 months).

Before propensity score matching, the median tumor height (8 cm [IQR 6–10 cm] vs 6 cm [IQR 4–7 cm], *p* = 0.000) and anastomotic height (5 cm [IQR 3–6 cm] vs 3 cm [IQR 1–4 cm], *p* = 0.000) were higher in the robotic group than the transanal group. Neoadjuvant chemoirradiation was performed in 38% patients in the RaTME group and 50% in the TaTME group (*p* = 0.115). Other demographic data including age, gender, body mass index, and tumor characteristics were comparable between groups before adjustment. After propensity score matching with tumor height and neoadjuvant irradiation being the independent variables, 74 matched pairs were included for further analysis. All baseline demographic and clinical parameters were comparable between the two groups after matching except more anastomoses were performed by handsewn manner in the transanal group (*p* = 0.005) (Table 1).

**Table 1:** Demographics and operative and pathological details after propensity score matching

	Robotic (*n* = 74)	Transanal (*n* = 74)	*p* value
Age	67.5 (30–89)	64.5 (45–84)	0.261
Male gender	56 (75.7%)	59 (79.7%)	0.693
BMI (kg/m^2^)	23.5 (15.9–31.1)	23.9 (15.9–30.6)	0.457
ASA grade			0.466
1	4 (5.4%)	3 (4.1%)	
2	47 (63.5%)	54 (73%)	
3	23 (31.1%)	17 (23%)	
Follow-up (months)	46.1 (8.7–90.7)	46.0 (5.9–92.3)	0.311
Neoadjuvant radiotherapy	32 (43.2%)	32 (43.2%)	1.0
Neoadjuvant chemotherapy	32 (43.2%)	33 (44.6%)	1.0
Adjuvant chemotherapy	46 (62.2%)	42 (56.8%)	0.787
Preoperative CEA (ng/ml)	4.9 (0.7–3498)	3.3 (0.6–570)	0.460
Tumor length (cm)	4.2 (1.1–10.9)	4.1 (0.9–7.7)	0.502
Tumor height (cm)	7 (2–10)	6 (2–10)	0.162
Anastomosis height (cm)	4 (1–8)	3 (1–7)	0.508
Type of anastomosis			0.005
Handsewn	2 (2.7%)	13 (17.6%)	
Stapled	72 (97.3%)	61 (82.4%)	
Intraoperative blood loss (ml)	100 (10–1300)	100 (10–1000)	0.526
Conversion to open surgery	1 (1.4%)	0 (0%)	1.0
Diversion stoma			0.880
Ileostomy	66 (89.2%)	66 (89.2%)	
Colostomy	4 (5.4%)	3 (4.1%)	
No stoma	4 (5.4%)	5 (6.8%)	
Time to closure (months)	8.6 (0.9–31.5)	8.4 (1.9–21.4)	0.771
Anastomotic leakage			0.193
Subclinical	5 (6.8%)	2 (2.7%)	
Symptomatic	0 (0%)	2 (2.7%)	
Anastomotic stricture	2 (2.7%)	1 (1.4%)	0.766
T stage			0.585
T1/2	25 (33.8%)	32 (43.2%)	
T3/4	45 (60.8%)	37 (50%)	
ypCR	4 (5.4%)	5 (6.8%)	
N stage			0.212
N0	43 (58.1%)	53 (71.6%)	
N1	27 (36.5%)	19 (25.7%)	
N2	4 (5.4%)	2 (2.7%)	
Tumor differentiation			0.680
Moderate	59 (84.3%)	59 (86.8%)	
Poor	5 (7.1%)	3 (4.4%)	
Mucinous	2 (2.9%)	1 (1.5%)	
Distal resection margin (mm)	22 (1.4–55)	15 (2–50)	0.131
Clear circumferential resection margin	73 (98.6%)	74 (100%)	0.500
Tumor recurrence	17 (23.0%)	14 (18.9%)	0.831
Local recurrence	5 (6.8%)	3 (4.1%)	
Distant recurrence	13 (17.6%)	12 (16.2%)	

### Anorectal functional assessment

**Fig. 1:**
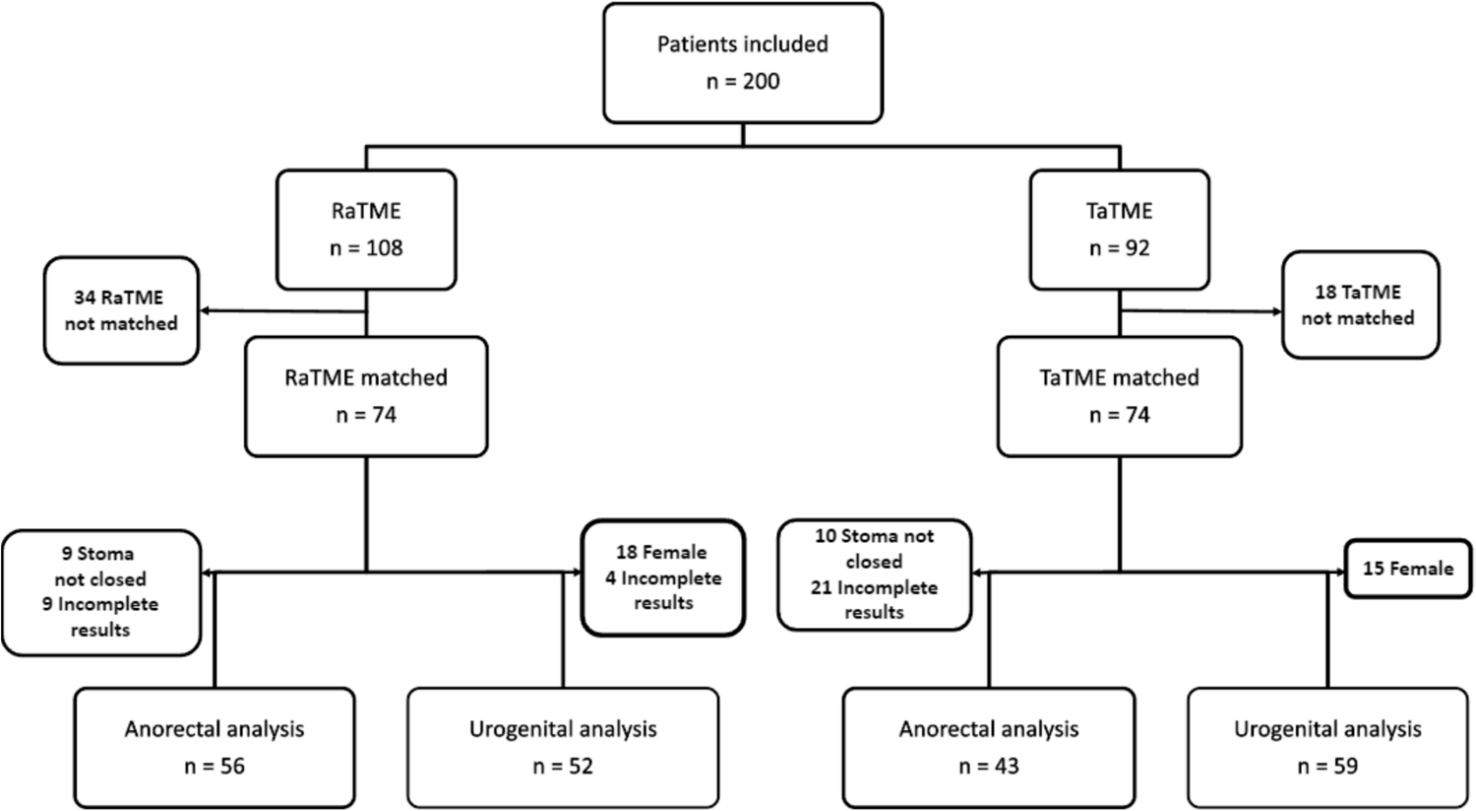
Flowchart of patient recruitment for functional assessment

During the index operation, the rate of diversion stoma was comparable between the RaTME and TaTME groups (70/74, 94.6% vs 69/74, 93.2%, *p* = 0.88). Diversion ileostomy or colostomy was performed in 89% and 5% of patients, respectively, while 9 patients (6%) had primary anastomosis without stoma. Stoma reversal was performed in 87.1% and 85.5% of patients in the RaTME and TaTME group, respectively, at a similar median time interval from initial procedure (8.6 vs 8.4 months, *p* = 0.771) (Table 1). Nineteen patients did not receive stoma closure by the end of study period as a result of various reasons including 10 patients with tumor recurrence, three patients with anastomotic stricture, and six patients had other medical comorbidities. Thirty patients declined questionnaire assessment. At 1 year after the index operation or stoma closure, 99 patients completed both questionnaires for anorectal function assessment and the overall response rate was 67% (Fig. 1).

#### LARS

**Fig. 2:**
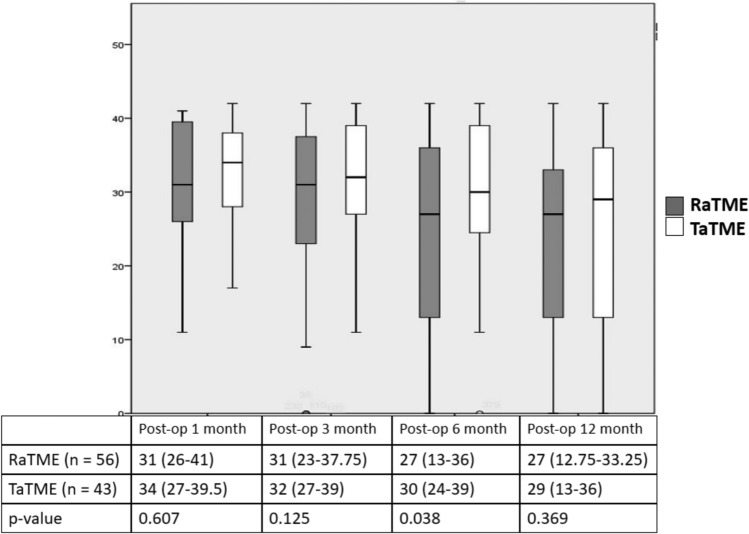
Anorectal function: box plot of LARS scores in RaTME and TaTME

The median LARS scores were lower in the RaTME group than in the TaTME group across the follow-up period while a significant difference was observed at postoperative 6 months (27 [IQR 13–36] vs 30 [IQR 24–39], *p* = 0.038). The difference in LARS scores between two groups converged at 1 year after operation (27 [IQR 13–33] vs 29 [IQR 13–36], *p* = 0.369) (Fig. 2). The severity of LARS in terms of rate of major LARS was similar in the robotic and transanal groups within the first postoperative year. The rate of major LARS, which was defined as LARS score of 30 or above, dropped from 65.2% and 64.7% at post-closure 1 month (*p* = 0.616) to 35.2% and 46.3% at post-closure 12 months (*p* = 0.297) in the robotic and transanal group, respectively. Although the rates of major LARS were relatively higher in the transanal group, no statistical differences were observed (Table 2).

**Table 2:** Severity of LARS in RaTME and TaTME

Postoperative follow-up	RaTME (*n* = 56)	TaTME (*n* = 43)	*p* value
1 month			0.616
No LARS	8.7%	11.8%	
Minor LARS	26.1%	23.5%	
Major LARS	65.2%	64.7%	
3 months			0.405
No LARS	16.1%	11.6%	
Minor LARS	26.8%	20.9%	
Major LARS	57.1%	67.4%	
6 months			0.416
No LARS	34.5%	18.6%	
Minor LARS	23.6%	30.2%	
Major LARS	41.8%	51.2%	
12 months			0.297
No LARS	35.2%	39%	
Minor LARS	29.6%	14.6%	
Major LARS	35.2%	46.3%	

#### Wexner incontinence score

**Fig. 3:**
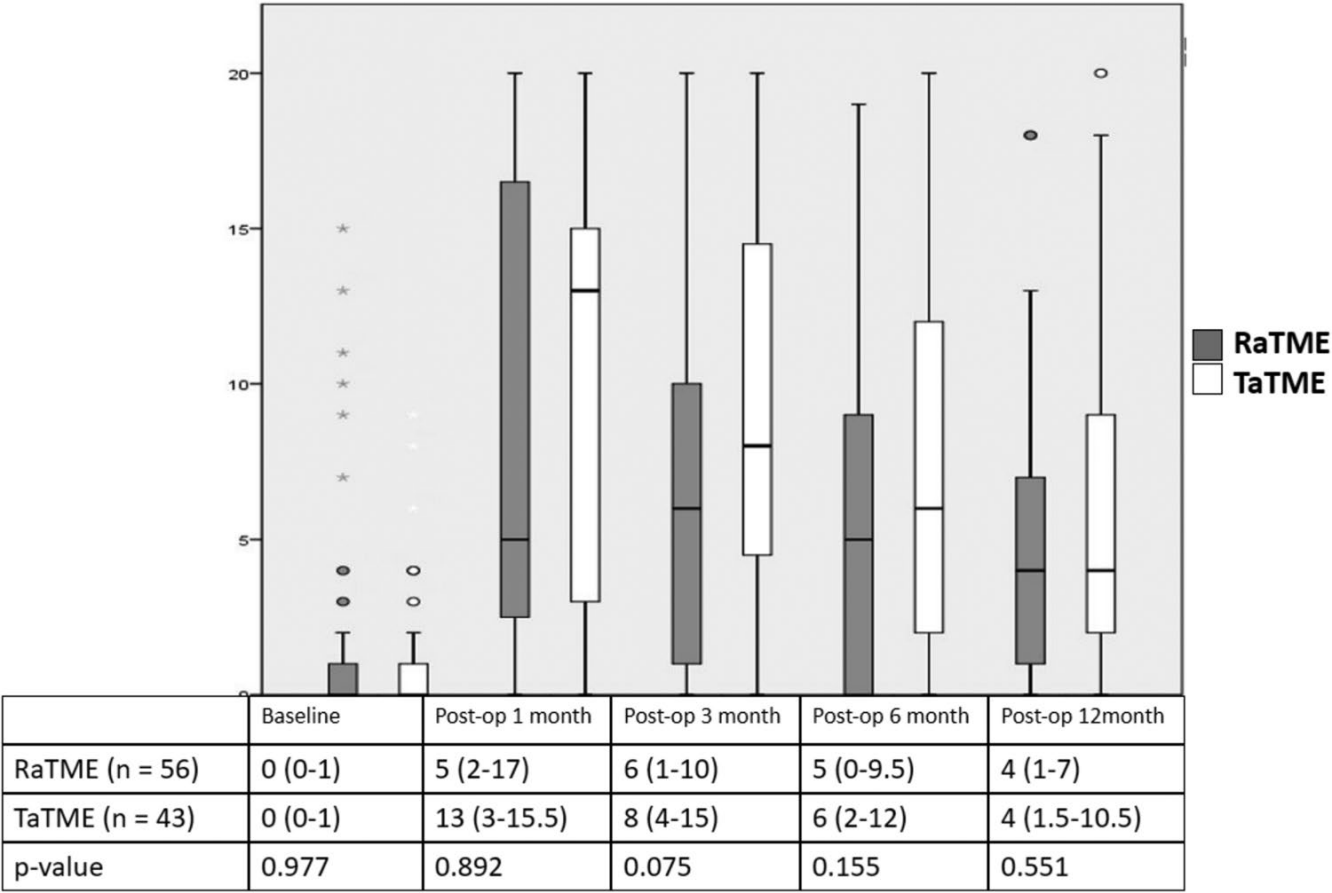
Anorectal function: box plot of Wexner incontinence scores in RaTME and TaTME

The RaTME group has lower Wexner incontinence score than the TaTME group at 6 months after stoma closure (5 [IQR 0–9.5] vs 6 [IQR 2–12], *p* = 0.155) but the difference was not statistically significant. The Wexner score rose from zero to a plateau (5 [IQR 2–17] vs 13 [IQR 3–16], *p* = 0.892) at postoperative first month then gradually decreased in both the RaTME and TaTME group. This pattern of score change was observed in both groups similarly. The median Wexner scores were relatively higher in the TaTME group over the study period, though the differences did not reach significance. Despite a gradual recovery over time, patients in both groups still experienced residual compromise in bowel continence at 1 year after stoma closure when compared with preoperative scores (Fig. 3).

#### Subgroup analysis

Subgroup analysis of anorectal function between patients with and without diversion stoma was performed. The LARS scores were relatively lower in patients without stoma across the postoperative period from 1 month to 12 months. However, statistical significance was unable to be achieved as a result of the small sample size. Similarly, lower Wexner scores were observed favoring patients without protective stoma (Table 3).

**Table 3:** Comparison of postoperative LARS and Wexner scores between patients with and without diversion stoma at 1 to 12 months

	Diversion stoma (*n* = 139)	No stoma (*n* = 9)	*p* value
LARS score			
1 month	32 (26–40.25)	29.5 (20–39)	0.662
3 months	32 (24–39)	27 (21–36.5)	0.872
6 months	29 (21–38.5)	20 (11.5–35.5)	0.657
12 months	27.5 (13–34)	23 (5–33.5)	0.548
Wexner score			
1 month	9.5 (3–16)	7 (0–14)	0.813
3 months	7 (3–12)	6 (1–10.5)	0.294
6 months	6 (2–10)	1 (0–7.5)	0.182
12 months	4 (1.75–9)	2 (0–3.5)	0.092

To evaluate the effect of duration of intestinal diversion on functional recovery, subgroup analysis was performed between patients with early stoma closure within 8 months versus late closure beyond 8 months after index operation. The LARS score was significantly lower in the early closure group (30 [IQR 24.5—35]) than in the late closure group (37 [IQR 29–41]) at 1 month after closure (*p* = 0.043). At 3 months to 12 months after closure, LARS scores remained relatively lower in the early closure group but the differences were diminished. No significant differences in Wexner scores were seen between the two groups (Table 4).

**Table 4:** Comparison of postoperative LARS and Wexner scores at 1 to 12 months between patients with early and late stoma closure

	Early closure, ≤ 8 months (*n* = 56)	Late closure, > 8 months (*n* = 64)	*p* value
LARS score			
1 month	30 (24.5–35)	37 (29–41)	0.043
3 months	31.5 (24.75–39)	32 (23–39)	0.920
6 months	28 (16–39)	30 (22.25–37)	0.431
12 months	27 (13–36)	29 (16.25–33.25)	0.829
Wexner score			
1 month	4 (1–14)	13 (3–17)	0.522
3 months	7 (2.5–10.5)	7 (3–13)	0.865
6 months	4 (2–10)	7 (2–10)	0.414
12 months	4 (1–9)	5 (2–9.25)	0.362

### Urogenital functional assessment

In the matched RaTME and TaTME subgroups, 18 and 15 female patients were excluded from urogenital functional assessment, respectively. Four patients in the RaTME group did not complete questionnaire assessment while the response rate in the TaTME group was 100%.

#### IPSS

**Fig. 4:**
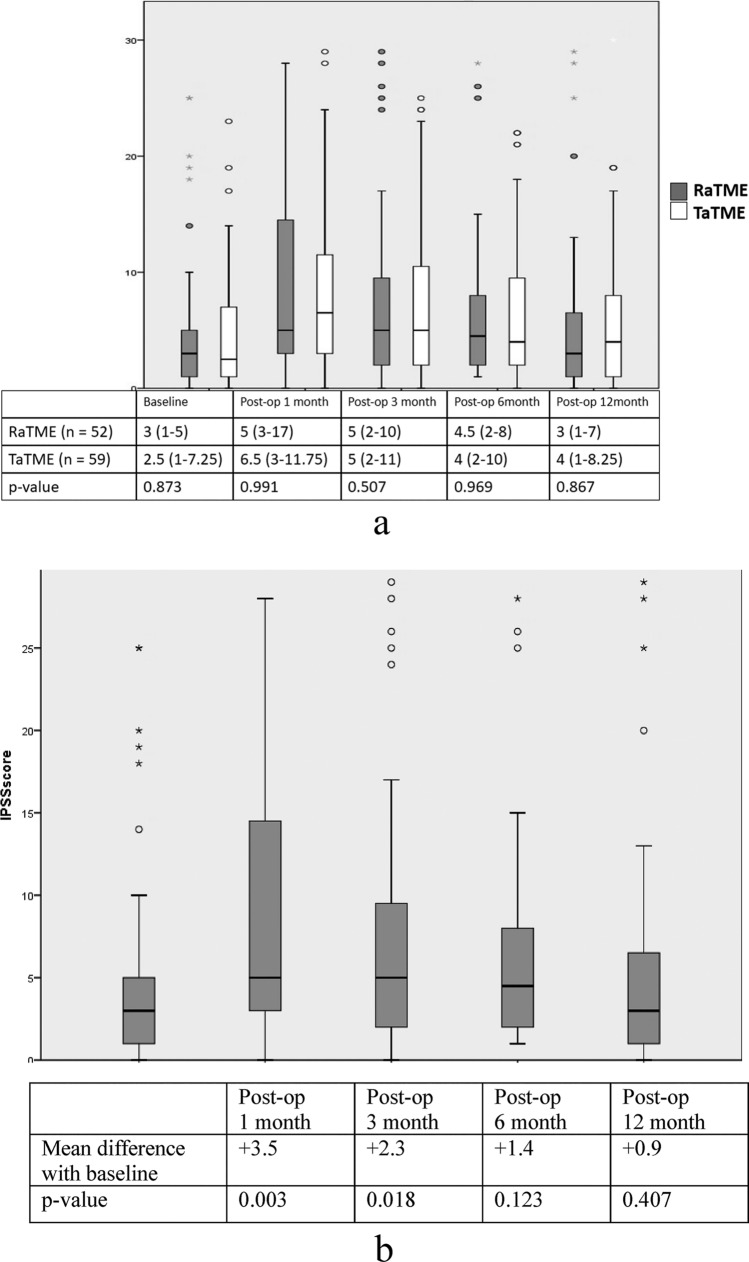

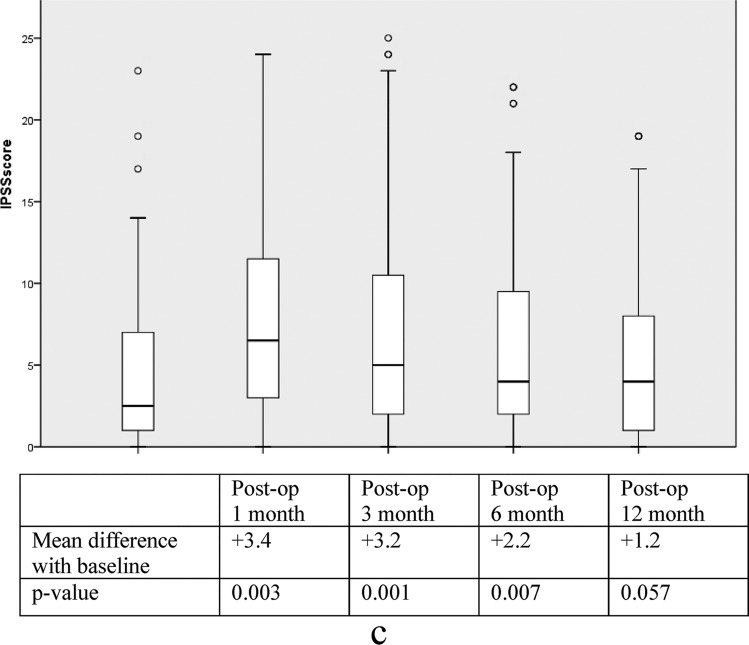
Urinary function: **a** box plot of IPSS in RaTME and TaTME. **b** Comparison of IPSS with baseline in RaTME. **c** Comparison of IPSS with baseline in TaTME

The serial changes of IPSS in both groups shared a similar pattern as described in Wexner incontinence score in which deterioration was seen at the first month followed by a gradual return to baseline. No significant intergroup difference was observed again (Fig. 4a). In subgroup analysis by paired sample *t* test, serial scores at postoperative 1, 3, 6, and 12 months were compared with preoperative scores. The maximum paired difference was demonstrated at the first month with an IPSS score increment of 3.5 in the RaTME group (*p* = 0.003) and 3.4 in the TaTME group (*p* = 0.003). The recovery of urinary function occurred earlier in the RaTME group and the significant paired difference disappeared at 6 months (*p* = 0.123) (Fig. 4b). On the other hand, a delayed return was observed at 12 months in the TaTME group (*p* = 0.057) (Fig. 4c). The quality-of-life scores due to urinary symptoms did not change significantly over the follow-up period nor varied between the two groups.

#### IIEF

**Fig. 5:**
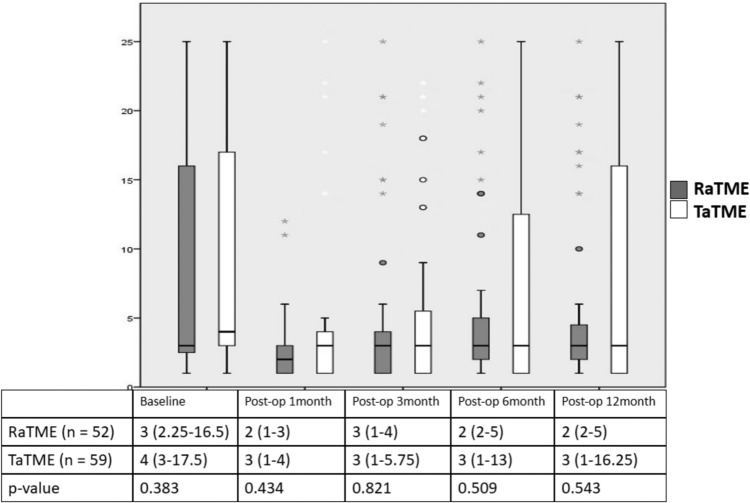
Sexual function: box plot of IIEF-5 in RaTME and TaTME

Among all included male patients in this cohort, 42% were sexually active before the index operation. The proportion of sexually active male patients dropped to 18% at the first postoperative month and was up to 28% at 1 year. In sexually inactive male patients, only the first item in the IIEF questionnaire was scored and the rest was scored zero. The scores in both groups demonstrated homogenous serial change with a fall in first month followed by a gradual return. There was no statistical difference in the IIEF scores between the two groups over the study period (Fig. 5).

### Oncological outcome and safety

**Fig. 6:**
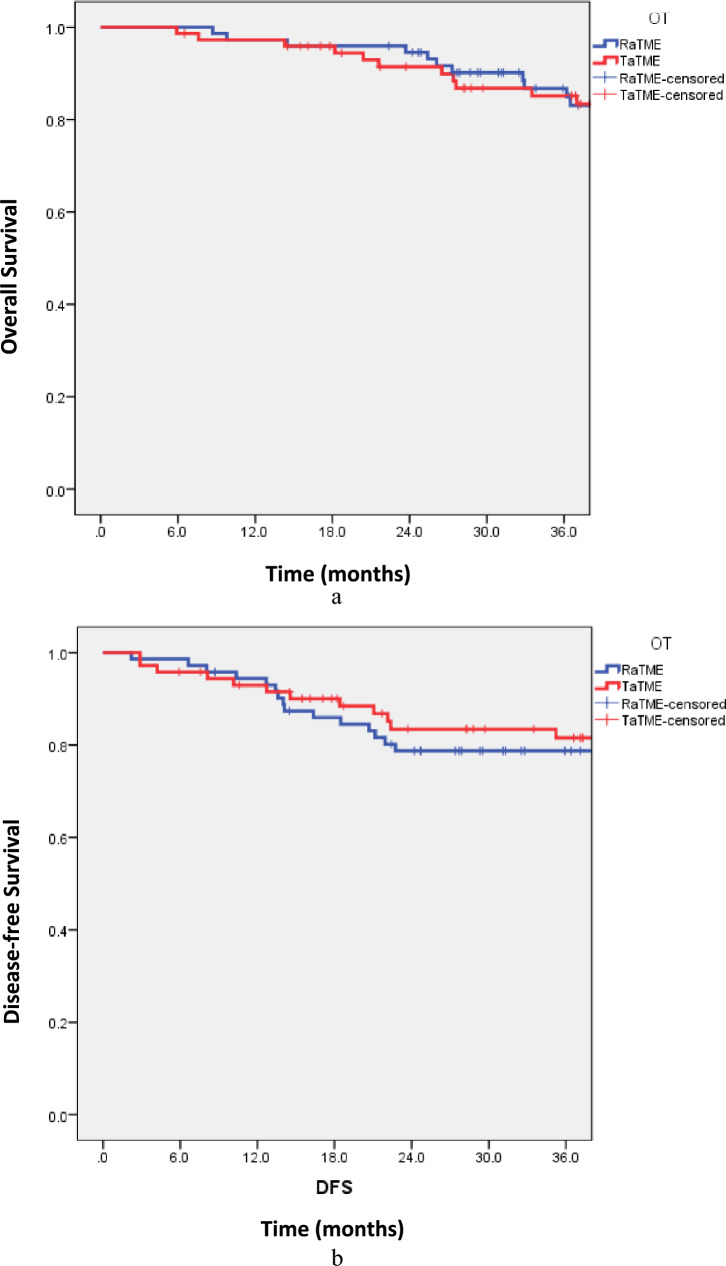
Three-year **a** overall survival and **b** disease-free survival of RaTME and TaTME

The oncological outcome and safety were comparable between the two approaches. The 3-year overall survival was 87.8% in RaTME and 86.5% in TaTME (hazard ratio 0.86 [95% confidence interval 0.43–1.71], *p* = 0.663) (Fig. 6a). Three-year disease-free survival was 79.7% in RaTME and 83.8% in TaTME (hazard ratio 1.10 [95% confidence interval 0.54–2.24], *p* = 0.789) (Fig. 6b). Local recurrence was found in 6.8% and 4.1% of RaTME and TaTME patients, respectively (*p* = 0.831). Only one patient who underwent RaTME (1.4%) was converted to open surgery because of intraoperative bleeding while none in the TaTME group required conversion.

## Discussion

Functional preservation after TME has gained much attention over the past few years [19]. Despite the growing popularity of both robotic and transanal TME, direct comparative studies of the functional outcome between these two approaches were scarce. This is the first propensity score-matched prospective study evaluating both anorectal and urogenital outcome in RaTME and TaTME patients. We demonstrated a superiority of RaTME in preserving anorectal function in terms of lower LARS score and earlier recovery of urinary function at postoperative 6 months.

The debate about top-down versus bottom-up TME approach has been unresolved since the introduction of TaTME in 2009 [20]. According to previously published results from our center, TaTME was associated with higher median LARS score of 37 at the initial 3 months (*p* = 0.045) but the difference disappeared in the long run [13]. Alimova et al. also showed higher LARS scores after TaTME (weighted mean difference 2.88; 95% confidence interval 0.15–5.60; *p* = 0.04) but similar long-term oncological outcome and quality of life between laparoscopic and transanal TME in a systematic review of 10 observational studies [11]. However, the differences in anorectal and genitourinary outcomes were not reproduced by another meta-analysis [12].

Whether functional outcomes were better preserved after a bottom-up approach remained controversial [21]. On the one hand, better visualization and protection of pelvic nerves intraoperatively during transanal dissection was hypothesized [22]. On the other hand, prolonged anal dilatation by insertion of transanal platform and subsequent sphincter muscle injury was postulated to jeopardize defecatory function [23]. An attempt to establish objective correlation between bowel function and manometric data was reported. However, only a significant decline in squeeze pressures between pre- and postoperative manometry was confirmed (*p* = 0.003). No major difference between laparoscopic and transanal approach was seen [24].

Regarding the abdominal dissection part, use of robotic assistance over conventional laparoscopic surgery has been supported by various trials with encouraging clinical and oncological outcomes [25]. Eltair et al. evaluated nine randomized controlled trials and suggested robotic TME was associated with significantly longer distal resection margin (*p* = 0.004) and shorter time to soft diet (*p* = 0.03) when compared with laparoscopic TME despite longer procedure time (*p* = 0.002) and similar LARS (*p* = 0.41) [26]. A systematic review comparing functional outcomes between all four TME techniques, namely open, laparoscopic, robotic, and transanal TME, demonstrated that RaTME outperformed the other three techniques in terms of lower rate of LARS (*p* = 0.003). However, this review only included one study regarding robotic approach and most of the studies were case series only [27]. In terms of urogenital function, a matched analysis echoed our findings of early functional recovery at postoperative 6 months in the robotic group when compared to recovery at postoperative 12 months in the laparoscopic group [28].

The advantage of robotic surgery over laparoscopic surgery was attributed to the high-definition camera and articulated instruments which allowed visualization and preservation of autonomic nerve plexus in the deep pelvis [29]. With nerve injury being minimized by meticulous dissection, the effect on bowel and urogenital dysfunction was reported to be self-limiting and reversible within the first year after operation when local inflammation subsided [30].

Given the characteristic benefits of both transanal and robotic TME, a direct comparison between the clinical outcomes of these two approaches would be crucial [31]. Grass et al. conducted an observational study comparing functional outcomes between robotic and transanal TME in 120 patients [32]. The anorectal function was shown to be superior in the robotic group than the transanal group in terms of lower LARS score (4.3 ± 2.2 vs 9.8 ± 1.5, *p* = 0.038) but the urinary function was better preserved in male patients in the transanal group (ICIQ-MLUTS: RaTME 13.8 ± 4.9 vs TaTME 1.8 ± 5.8, *p* = 0.038). This study was limited by the heterogeneity in surgical techniques and patient demographics among different participating centers. Another questionnaire-based study also showed that RaTME provided better bowel function but without significant difference and similar urinary function when compared with TaTME [33]. Seow et al. performed a network meta-analysis and did not show any difference in urinary and male sexual function among all four TME techniques [34]. To date, concrete evidence to compare functional outcomes between RaTME and TaTME was still lacking.

In this study, we showed that TaTME resulted in short-term deficit in anorectal function when compared to RaTME. The difference in LARS scores between the two groups became insignificant at 1 year after operation. Bjoern et al. and Li et al. similarly demonstrated a temporary decline in anorectal function and quality of life after TaTME followed by normalization after 1 year [9, 35]. The rates of major LARS in our series lowered to 35.2% in RaTME and 46.3% in TaTME at 1 year (*p* = 0.297). These findings were in concurrence or even lower than the rate of major LARS (47.9–53.3%) published in a previous meta-analysis [12].

Based on the phenomenon that functional disturbance was reversible but more pronounced after TaTME, early intervention could be beneficial to this group of high-risk patients. Early stoma closure has been suggested to prevent disuse colitis and preserve anorectal function by Tirelii et al. [36]. The benefit of early closure within 8 months was proven in our subgroup analysis as evidenced by a significantly lower LARS score at postoperative 1 month. It should be considered especially in TaTME patients as soon as anastomosis was confirmed intact in order to expedite functional recovery. Besides, the importance of perioperative patient education and pelvic floor rehabilitation could not be overemphasized [37].

Regarding statistical analysis, propensity score matching was performed with tumor height and use of neoadjuvant radiotherapy being adjusted. According to previous studies, tumor location and presence of radiotherapy were both significant predictors affecting bowel and urogenital function postoperatively [19, 38]. Since TaTME was designated to resect more distally located rectal tumor, discrepancy in bowel continence between the two groups would be unavoidable before matching owing to differences in the anastomotic height and residual rectal volume. Matching was essential to establish a fair comparison between RaTME and TaTME. After patient and tumor characteristics were well matched, we believed the technical impact of each approach could be reflected without bias.

Nevertheless, there were a few limitations in our study. First, 18 and 15 female patients were excluded from urogenital assessment in the RaTME and TaTME group, respectively. The small number of female patients precluded a powered subgroup analysis for functional scores. Further studies focusing on female urogenital function would be valuable although previous published literature suggested no significant difference between pre- and postoperative urinary [28, 30] and sexual dysfunction in female patients [19]. In addition, the percentage of sexually active men was low in our locality. This difference compared with other published series in western countries might be attributed to introverted culture in the Chinese population and patient embarrassment during interview with the questionnaire. The reported rate of sexual activity in our cohort was 28% at postoperative 1 year which was much lower than around 50% in another study by a Korean group [28]. The exact IIEF scores could be underestimated and the overall sexual function assessment was limited by reporting bias. Finally, the reported functional scores were up to postoperative 1 year in current review. Despite a return of function in terms of LARS and IPSS, Wexner incontinence scores and IIEF scores were not completely normalized at 1 year in both groups. Extended follow-up of functional performance in our patients beyond 1 year was ongoing and long-term bowel and sexual sequelae shall be addressed in future studies.

After all, functional outcome should only be explored in a background of preserved oncological safety which remained the foremost priority in managing patients with cancer. Substantial concerns regarding TaTME have been raised previously since the report of high local recurrence by a Norwegian group (7.9%) [39] and Dutch group (10%) [40]. Recently, the TaLaR study was published by a Chinese group which was the largest randomized controlled trial with more than 1100 patients comparing long-term oncological outcomes between transanal and laparoscopic TME [41]. Three-year disease-free survival for TaTME was shown to be non-inferior to that of laparoscopic TME. Postoperative 2-year functional outcome is planned to be investigated. A few other randomized controlled trials comparing oncological and functional outcomes between different approaches to TME were ongoing [42, 43]. Results from the COLOR III trial were planned to be published in the coming year. Long-term data from these studies would be warranted to verify the overall performance of different TME techniques.

## Conclusion

Robotic TME achieved better anorectal function with lower LARS score and earlier recovery of urinary function than transanal TME at postoperative 6 months. For long-term bowel and urinary function after 1 year, both groups performed similarly. Sexual function was comparable between the two groups in male patients.

## Data Availability

No datasets were generated or analysed during the current study.
